# Crystal structure, Hirshfeld surface analysis and DFT studies of 5-(adamantan-1-yl)-3-[(4-chlorobenzyl)sulfanyl]-4-methyl-4*H*-1,2,4-triazole, a potential 11β-HSD1 inhibitor

**DOI:** 10.1038/s41598-019-56331-z

**Published:** 2019-12-24

**Authors:** Lamya H. Al-Wahaibi, Jacques Joubert, Olivier Blacque, Nora H. Al-Shaalan, Ali A. El-Emam

**Affiliations:** 1 Department of Chemistry, College of Sciences, Princess Nourah bint Abdulrahman University, Riyadh, 11671 Saudi Arabia; 2 Pharmaceutical Chemistry, School of Pharmacy, University of the Western Cape, Private Bag X17, Bellville, 7535 South Africa; 3 Department of Chemistry, University of Zurich, Winterthurerstrasse 190, 8057 Zurich, Switzerland; 4 Department of Medicinal Chemistry, Faculty of Pharmacy, University of Mansoura, Mansoura, 35516 Egypt

**Keywords:** Computational chemistry, Medicinal chemistry

## Abstract

5-(Adamantan-1-yl)-3-[(4-chlorobenzyl)sulfanyl]-4-methyl-4*H*-1,2,4-triazole (**4**) was identified as a potential 11β-hydroxysteroid dehydrogenase type 1 (11β-HSD1) inhibitor and this paper describes the in-depth structural analysis thereof. Compound **4** was synthesized in a 92% yield and its 3D-structure confirmed by single-crystal X-ray diffraction. Hirshfeld surface analysis indicated that H^…^H, C-H^…^C, C-H^…^Cl and especially C-H^…^N hydrogen bond interactions are the primary contributors to the intermolecular stabilisation in the crystal. In order to explore the properties of **4**, free from the influence of the crystal field, density functional theory (DFT) calculations were conducted. Results indicated that the DFT optimized geometry of **4** produced a conformer (**4a**) that is significantly different from the crystal structure. Further experiments confirmed that the crystal structure is not the absolute minimum conformation. This indicated that the crystal packing forces has significantly influenced the conformation thereof. Frontier molecular orbital energies and net atomic charges were also calculated to elucidate the electronic properties of **4a**. These results provided insight into areas of the molecule that may present with the ability to form binding interactions at the 11β-HSD1 active site. Molecular docking experiments revealed important intermolecular interactions between **4a** and 11β-HSD1. These results indicate that **4** may be considered for further drug design endeavors.

## Introduction

**Figure 1:**
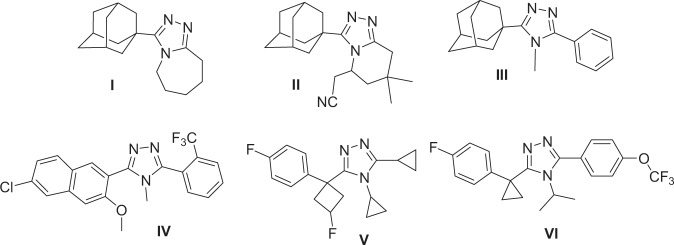
The structures the adamanty l-1, 2, 4-triazoles (**I**–**III**) and related clinical 1, 2, 4-triazoles (**IV**–**VI**) 11β-HSD1 inhibitors.

The insertion of an adamantyl moiety into various bioactive molecules leads to compounds with relatively higher lipophilicity, which in turn results in modification of their bioavailability and modulates their therapeutic indices^1–3^. Several adamantane-based drugs are currently used as efficient antiviral^4–7^, anti-TB^8,9^ and anticancer^10,11^ agents. In addition, vildagliptin^12^ and saxagliptin^13^ are currently used as oral hypoglycemic adamantane-based agents for the treatment of type 2 diabetes acting *via* inhibition of dipeptidyl peptidase IV. In 2003, the adamantyl-1,2,4-triazoles **I**, **II** and **III** were discovered as potent inhibitors of 11β-hydroxysteroid dehydrogenase type 1 (11β-HSD1)^14,15^. Currently a number of structurally related 1,2,4-triazole 11β-HSD1 inhibitors; **IV**^16^, **V**^17^, and **VI**^18^, are under clinical investigation (Fig. 1). The 11β-HSD1 enzyme is involved in the development of non-insulin-dependent diabetes, hyperglycemia, obesity, insulin resistance, hyperlipidemia, hypertension and other symptoms associated with excessive body cortisol^19–22^. Therefore, inhibitors of this enzyme could lead to the development of important therapeutic agents.

In continuation of our on-going interest in the structural studies and biological application of adamantane derivatives^23–27^, we report herein the crystal structure, Hirshfeld surface analysis, density functional theoretical studies and electronic properties of the title adamantane-triazole hybrid derivative **4**. In addition, compound **4** has a number of structural similarities to the 11β-HSD1 inhibitors shown in Fig. 1. Therefore, molecular docking experiments at the 11β-HSD1 active site were conducted in order to predict the potential 11β-HSD1 binding affinity and binding interactions of **4**.

## Results and Discussion

### Chemical synthesis

**Figure 2:**
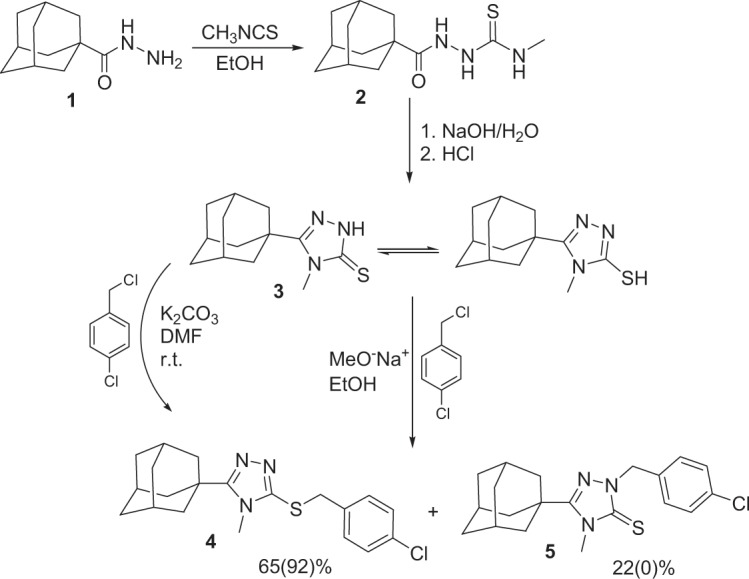
Synthetic approach for compound **4** (the percentage yields shown in parentheses are those obtained on carrying out the reaction in DMF with anhydrous potassium carbonate at room temperature).

The synthesis of compound **4** (Fig. 2) commenced from the reaction of adamantane-1-carbohydrazide **1** with methyl isothiocyanate yielding the corresponding thiosemicarbazide derivative **2**, which was cyclized to its 1,2,4-triazole analogue **3**. In the next step, **3** was reacted with 4-chlorobenzyl chloride in the presence of sodium methylate, in ethanol, at reflux temperature as previously described^28^. This method yielded the title compound **4** as a major product (65%) in addition to the undesirable *N*-chlorobenzyl analogue **5** as a minor product (22%). In an attempt to improve the yield of **4** and prevent the formation of **5**, different reaction conditions were studied at the 4-chlorobenzyl chloride conjugation step. We found that the use of *N*,*N*-dimethylformamide (DMF) as solvent, anhydrous potassium carbonate as base, with the reaction conducted at room temperature for 6 hours, led to the formation of compound **4** with a yield of 92% and the formation of the *N*-chlorobenzyl analogue (**5**) was avoided (Fig. 2).

### Crystal structure

**Figure 3:**
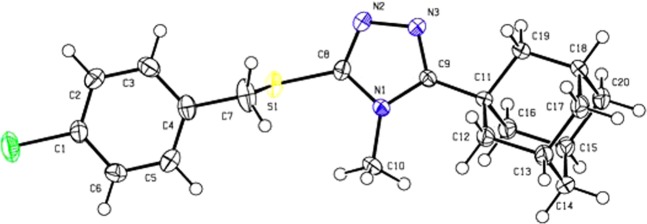
ORTEP diagram of the asymmetric unit of compound **4** drawn at 50% ellipsoids for non-hydrogen atoms.

**Figure 4:**
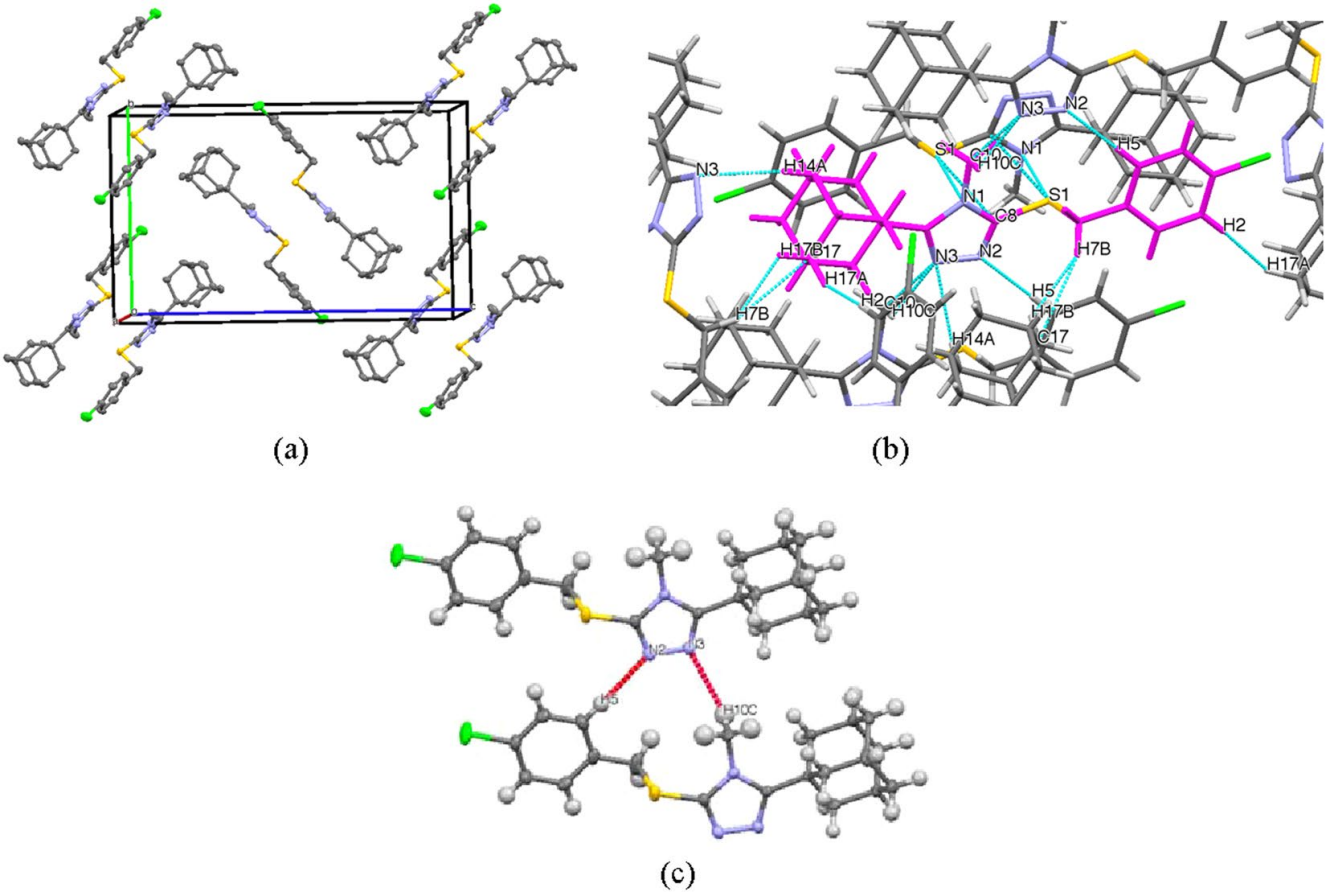
(**a**) Molecular crystal packing showing the head-to-tail dimers of compound **4** within the unit cell. (**b**) Illustration showing the complex series of short-range intermolecular interactions (cyan) within the crystal field. The molecule shown in magenta was used as the central point to illustrate all interactions present. (**c**) Schematic of the prominent non-classical C-H^…^N hydrogen-bonds (red) observed, as described in Table 2.

Crystals of compound **4** suitable for single crystal X-ray analysis were grown *via* slow evaporation of an ethanolic solution at room temperature. The ORTEP diagram along with the atom-labelling scheme is given in Fig. 3. X-ray crystallographic analysis revealed that **4** crystallizes in the monoclinic space group *P*2_1_/n, ß = 96.361(1)° and Z = 4 (full crystallographic data are presented in the materials and methods section. Selected bond lengths and bond angles of the adamantane ring, triazole-sulfanyl- and the chlorophenyl moiety are given in Table 1 and, in general, these parameters are comparable to other previously reported similar crystal structures^24–27^ (see the Supplementary material for a complete list of crystallographic parameters). The crystal packing arrangement shows the formation of pairs of dimers in an inversion related alternate head-to-tail molecular formation in the bc plane (Fig. 4a). Prominent non-classical C-H^…^N hydrogen-bonds are observed between the dimers and adjacent molecules (Table 2, Fig. 4c). Another non-prominent intramolecular C-H^…^N hydrogen bond is present between H14A of the adamantane moiety and N3 with an H^…^A distance of 2.652 Å and an angle of 123.57°. These hydrogen bonds add to the potential stability of the molecules within the crystal field and enables the formation, in addition to other short range contacts (Fig. 4b), of the three-dimensional network structure.

**Table 1:** Selected bond lengths (Å) and angles (°) of the crystal structure of compound **4**.

Bond	Length (Å)	Bond	Angle (°)
C1-C2	1.373(3)	C2-C1-Cl1	119.39(15)
C4-C7	1.505(3)	C5-C4-C7	119.51(19)
C7-S1	1.832(2)	C4-C7-S1	107.11(13)
C8-S1	1.752(17)	N1-C8-S1	125.05(13)
C8-N1	1.370(2)	N2-C8-N1	110.83(14)
C9-N3	1.316(2)	C9-C11-C12	111.98(13)
C10-N1	1.464(2)	C20-C18-C17	109.56(15)
N2-N3	1.380(2)	C8-N1-C9	104.71(13)
C9-C11	1.505(2)	C9-N2-N3	106.74(14)
C11-C19	1.546(2)	C9-N3-N2	108.40(13)
C15-C20	1.534(3)	C8-S1-C7	102.14(8)

**Table 2:** Prominent hydrogen bonds for **4**.

D-H^…^A	d (H^…^A)/Å	d (D^…^A)/Å	(D-H^…^A)/°
C5-H5^…^N2^1^	2.41	3.353 (2)	175.6
C10-H10C^…^N3^i^	2.59	3.215 (2)	121.9

Symmetry codes: (i) –x + 1, −y + 1, −z + 1.

### Hirshfeld surface analysis

**Figure 5:**
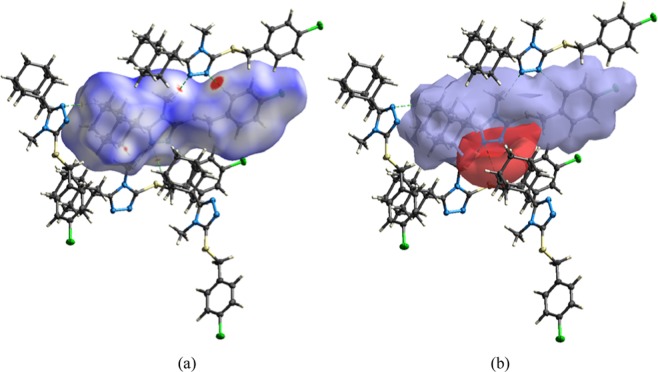
(**a**) The three-dimensional Hirshfeld surface showing the intermolecular interactions of **4** plotted over d_norm_. (**b**) Electrostatic potential of **4** mapped using the 6-311 G(d,p) basis set at B3LYP level theory over a range ± 0.03 a.u. Dotted lines (green) signify prominent C-H^…^N hydrogen bonds.

The Hirshfeld surface and subsequent fingerprint plots were calculated to quantify the intermolecular contacts present within the crystal structure of compound **4**^29,30^. The respective acceptor and donor atoms showing strong C-H^…^N intermolecular hydrogen bonds (for C5—H5^…^N2, C10—H10C^…^N3 and C14-H14A^…^N3) are indicated as bright red spots on the Hirshfeld surface (Fig. 5a). This finding is substantiated by the calculated electrostatic potential (Fig. 5b) of the molecule that was used to generate the Hirshfeld surface. The negative potential (acceptor) is indicated as a red surface around the two nitrogen atoms (N2 and N3) and the blue surface area, indicating the positive potential (donor), is mapped in the proximity of the hydrogen atoms (H5, H10C and H14A).

**Figure 6:**
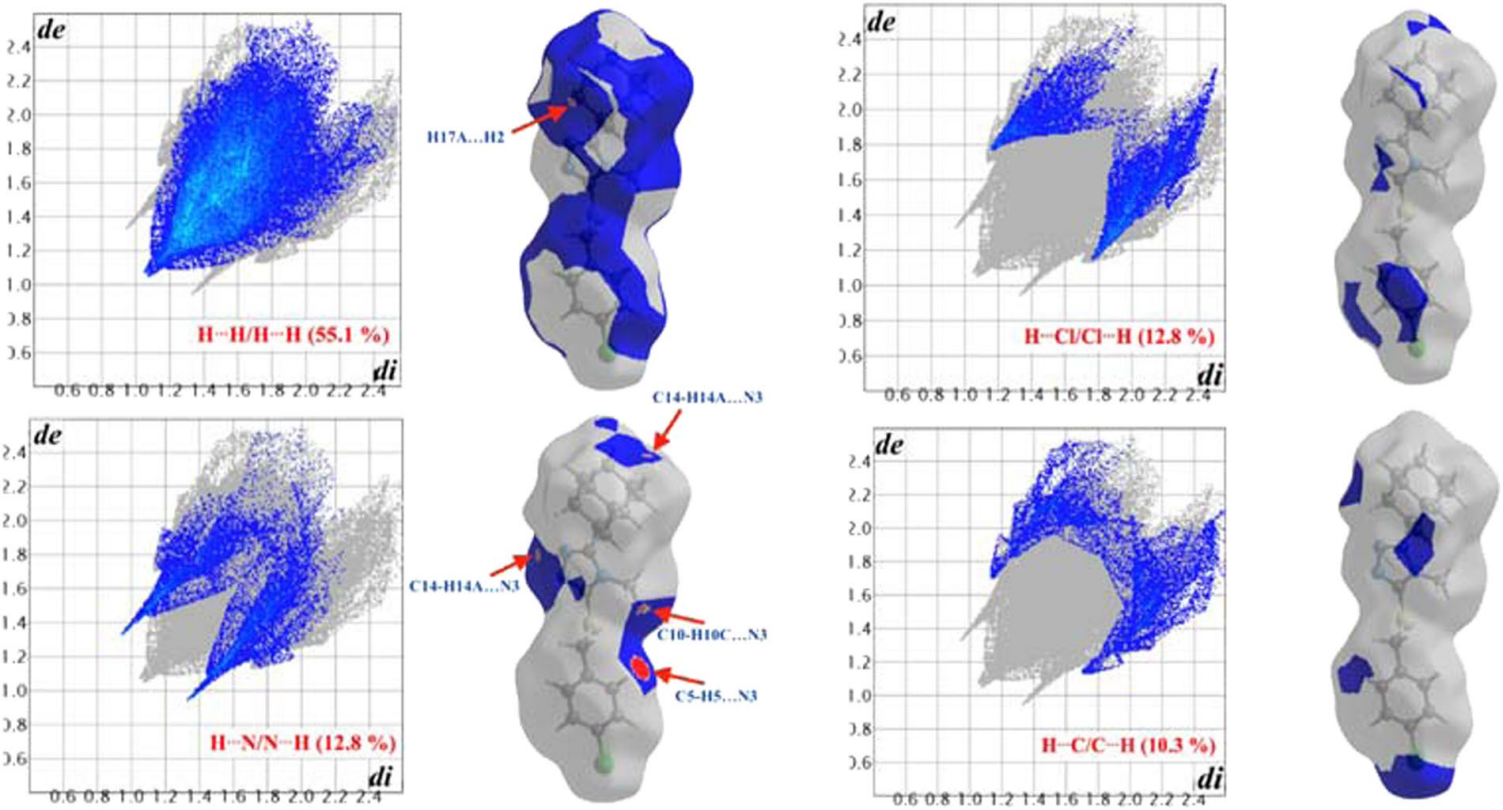
Fingerprint plots of **4**, showing the contributions of atoms within specific interacting pairs (blue areas). For each fingerprint map, the grey area is a representation of the whole plot. Surface maps next to each fingerprint plot indicate the applicable areas (indicated in blue) that are associated with the specific intermolecular contact(s).

**Figure 7:**
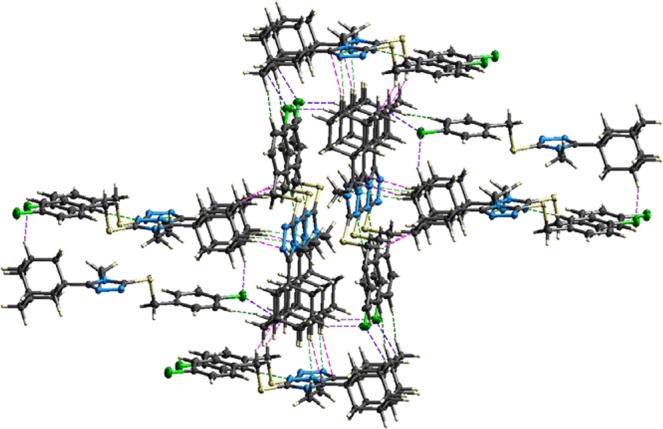
Partial packing diagram showing the most prominent intermolecular interactions present within the crystal structure of **4** based on the Hirshfeld surface analysis findings. Interaction color scheme: H^…^H = Red; CH^…^N = Green; C^…^H = Magenta; Cl^…^H = Blue.

Significant intermolecular interactions are mapped in Fig. 6. On the Hirshfeld surfaces the H^…^H interactions appear as the largest region (55.1%) of the fingerprint plot with a high concentration at *d*_*e*_ = *d*_*i*_ ~1.2 Å. It is of interest that the H^…^H bond between H2 and H17A is indicated as a red spot on the Hirshfeld surface indicating that this is a significant short range H^…^H contact. Two sharp spikes (*d*_*e*_ + *d*_*i*_ ~2.2 Å) on the fingerprint plot were observed for the N⋯H/H⋯N contacts, corresponding to the C—H^…^N interactions. These spikes are indicative of a strong hydrogen-bond interaction. The C^…^H/H^…^C contacts contribute to 10.3% of the Hirshfeld area. These contacts also appear as two spikes in the vicinity of *d*_*e*_ + *d*_*i*_ ~2.5 Å. Cl^…^H/Cl^…^H contacts (*d*_*e*_ + *d*_*i*_ ~3.6 Å), corresponding to C-H^…^Cl bond interactions, contribute to 12.8% of the surface area. This contact is however not visible as a red spot on the Hirshfeld surface. Most probably attributed to the fact that the sum of the van der Waals radii between the interacting C-H^…^Cl atoms is smaller than the interatomic distances between these atoms. All other contacts observed were found to contribute less than 4.3%. It is therefore clear that the H^…^H, C^…^H/H^…^C, Cl^…^H/Cl^…^H and especially N^…^H/H^…^N contacts, were the most significant contributors among the interacting atoms. This finding therefore indicates the significance of these contacts in the packing arrangement of the crystal structure. Based on these findings a detailed model was constructed showing the most prominent short range intermolecular contacts that are responsible for the packing arrangement and formation of the three-dimensional network structure of **4** (Fig. 7).

### Geometry optimization

Since we also wanted to investigate the potential 11β-HSD1 binding affinity of **4**, we were interested in the molecular conformation thereof released from the crystalline field and without the influence of the intermolecular contacts. After determining the compound’s three-dimensional structure in crystalline state, the next step was to define the energy minima conformation thereof. To do this, the Gaussian09 program package was used to perform a density functional theory (DFT) geometry optimization according to the methods as previously described^31–34^.

**Figure 8:**
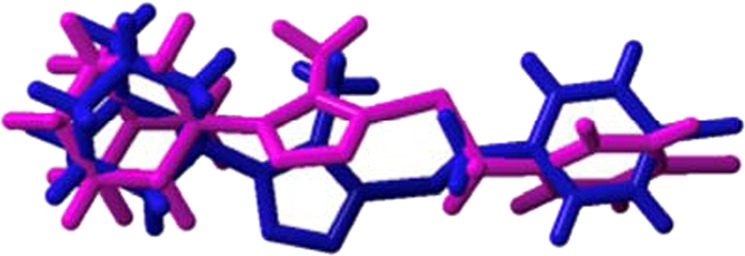
Atom-by-atom superimposition of the DFT optimized compound (**4a**, magenta) on the X-ray structure (blue) of the title compound (RMSD = 1.491 Å, performed using YASARA version 18.11.21 (YASARA Biosciences GmbH)).

The conformation of the molecule changed significantly in its DFT optimized state (**4a**) when compared to the crystal structure (Fig. 8). This is confirmed by comparing selected torsion angles of the molecule, obtained in the crystal structure analyses and after DFT optimization (Table 3). Table 3 shows that the DFT optimized N1-C8-S1-C7 dihedral angle is quantitatively closer to being flat. This could suggest that the sulfur π-π lone pair may be orthogonally oriented and possibly participates in a π-electron delocalization that is extended to the planar triazole ring leading to the observed DFT molecular minimum of **4a**. To verify this point, the torsion around C8-S1 was fixed orthogonally and the DFT experiment was repeated in order to generate a conformer (**4b**) that is very close (RMSD = 0.221 Å) to the one observed in the crystal structure (see Supplementary information Figs. S2 and S3). Comparison of the energy differences between **4a** (27.973 kcal mol^−1^) and **4b** (47.495 kcal mol^−1^) indicates that **4a** is a more stable system by as much as 19.5 kcal/mol. It is therefore confirmed that the experimental crystal structure is not the absolute minimum and that in the solid state the observed conformation is attributable to the crystalline packing forces. This phenomenon is similar to findings described in the literature^35,36^. The DFT optimized structure (**4a**) is therefore deemed the appropriate conformer for further structural investigation in order to determine the potential 11β-HSD1 binding affinity of **4**.

**Table 3:** Selected torsion angles (Å) of the crystal structure and the DFT optimized structure (**4a**).

Crystal structure	Crystal structure	DFT optimized structure^a^
Cl1-C1-C2-C3	179.12	−179.92
C3-C4-C7-S1	−85.68	−87.33
C4-C7-S1-C8	179.33	169.53
N2-C8-N1-C10	−177.78	177.56
S1-C8-N1-C9	177.28	175.58
S1-C8-N2-N3	−177.37	−174.80
N1-C8-S1-C7	82.82	−147.06
N3-C9-C11-C12	126.1	98.62
C11-C9-N1-C8	179.65	−178.58
N3-C9-N1-C10	177.85	−177.99
C9-C11-C12-C13	−178.43	−178.64
C19-C11-C12-C13	−59.54	−59.24

^a^The B3LYP/6-311++G(d,p) level of theory was used for optimization.

### Frontier molecular orbitals (FMO) and net atomic charges

**Figure 9:**
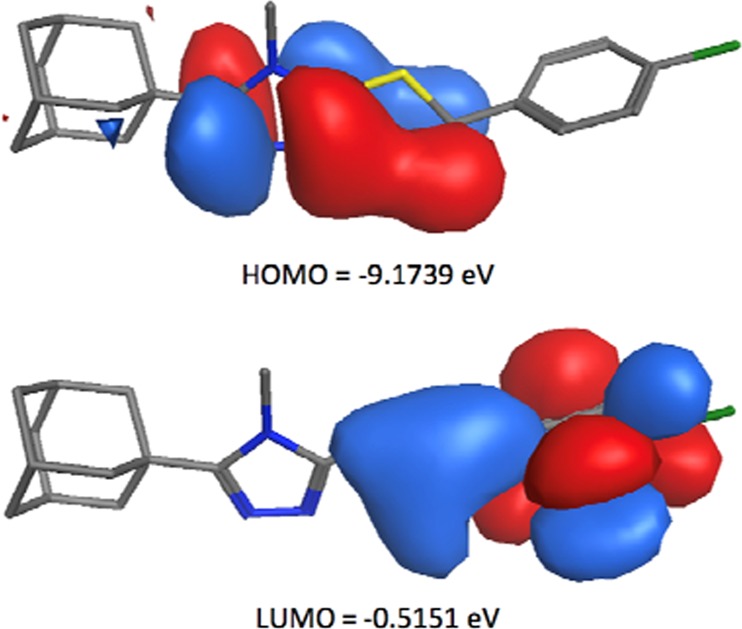
The HOMO and LUMO FMOs of conformer **4a** calculated using B3LYP/6-311++G (d,p).

Frontier orbital energy studies can provide valuable insights into biologically active compounds’ potential biological mechanisms^37–40^. Figure 9 shows the distribution and energy levels of the HOMO (highest occupied molecular orbital) and LUMO (lowest unoccupied molecular orbital) orbitals calculated for conformer **4a** at the B3LYP/6-311 G level. The electron distribution is mainly scattered in HOMO over the triazole ring and the sulfur moiety, whereas the LUMO is mainly disseminated over the sulfur- and chlorophenyl moieties. This indicates that there is a transfer of charge between the triazole ring and the chlorophenyl moiety. The Eigen values of LUMO (−0.5151 eV) and HOMO (−9.1739 eV) and their large energy gap (8.6588 eV), provides further support that a charge transfer exist within **4a** which could promote its bioactivity and ability to form biological interactions at the triazole- and/or chlorophenyl moiety. In addition, the low total energy and large energy gap of HOMO-LUMO suggest that the molecule has good stability and is in its lowest energy conformation.

**Figure 10:**
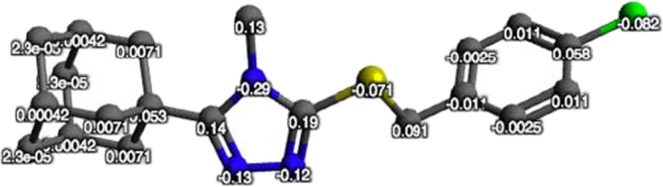
The net atomic charges (e) of the DFT optimized structure of compound **4**.

To study the electronic properties of **4a**, the net atomic charges of the DFT optimized structure were calculated using Avogadro 1.2.0 (Fig. 10). The three *N*-atoms in the triazole ring have the highest electronegativity and the lowest net atomic charge values (N1 = −0.29 e, N2 = −0.12 e and N3 = −0.13). It is therefore expected that the triazole moiety as a whole or the individual *N*-atoms contained within the structure will be the most favored to interact through hydrogen bond interactions and/or other intermolecular forces with closely positioned amino acids within the 11β-HSD1 active site. In addition, the sulfur and chlorine moieties also have relatively low net atomic charges (S = −0.071 e and Cl = −0.082 e) and may also be involved in productive intermolecular interactions or may aid the ability of functional moieties they are conjugated to, such as the phenyl moiety, to form binding interactions with the enzyme.

### Molecular docking studies

The Molecular Operating Environment (MOE) 2018 suite was used for the molecular docking experiments of compound **4**^41^. The crystal structure (Protein Data Bank I.D.: 4C7J) of human 11β-HSD1 co-crystallized with the 11β-HSD1 inhibitor 4-cyclopropyl-*N*-(trans-5-hydroxy-2-adamantyl)-2-(2-hydroxyethoxy)-thiazole-5-carboxamide (4YQ)^40^ was used for all the docking experiments. 4YQ has a potent 11β-HSD1 IC_50_ value of 9.9 nM and is structurally related to compound **4** of this study^42^. This enzyme and the co-crystallized ligand is therefore deemed appropriate to explore and predict the potential 11β-HSD1 inhibitory activity of compound **4**. The DFT optimized conformer (**4a)** was used for the docking experiments.

**Figure 11:**
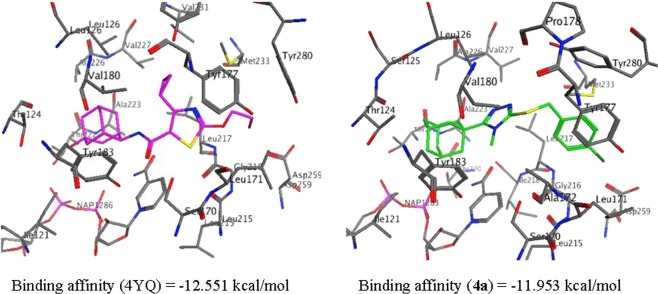
The binding mode, orientation and predicted binding affinity (kcal/mol) of 4YQ (magenta) and **4a** (green) within the 11β-HSD1 active site.

**Figure 12:**
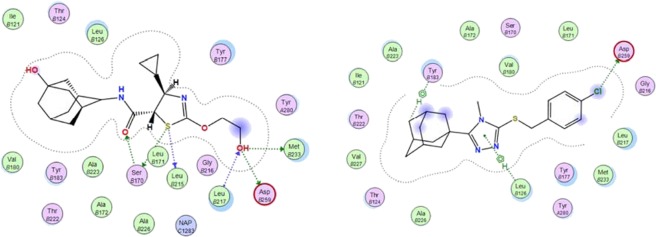
Binding interactions of the co-crystallized ligand, 4YQ (left) and conformer **4a** (right), within the 11β-HSD1 active site.

The binding mode of the co-crystallized ligand, 4YQ, indicated the formation of six hydrogen bond contacts through the interaction of the carbonyl, sulfur and hydroxyl moieties with residues Ser170, Leu215 and Asp259 (Figs. 11 and 12). These interactions are responsible for the potent inhibition of 11β-HSD1 by 4YQ (human 11β-HSD1 IC_50_ = 9.9 nM)^42^. The binding affinity of the co-crystallized ligand, 4YQ, was calculate as −12.511 kcal/mol. Using the protocol as described in the experimental section, the DFT optimized minimum energy conformer (**4a**) was docked. The results showed that **4a** was able to access and bind to the 11β-HSD1 active site, with an orientation similar to 4YQ (Figs. 11 and 12). Two H-π interactions between the adamantane and Tyr183 and the triazole and Leu126 were observed. In addition, a hydrogen bond interaction was observed between the halogen function and Asp259. These results were as expected based on the FMO and net atomic charge findings as discussed in the previous sections. The predicted binding affinity of **4a** was found to be −11.953 kcal/mol. These interactions, together with the predicted binding affinity, which is similar to the binding affinity of 4YQ, indicate that **4** should show some significant 11β-HSD1 inhibitory activity within a similar range as 4YQ. However, biological tests need to be done in order to validate the computational predictions.

## Conclusion

5-(Adamantan-1-yl)-3-[(4-chlorobenzyl)sulfanyl]-4-methyl-4*H*-1,2,4-triazole (**4**) was synthesised in a 94% yield using an optimized synthesis method. Single crystal X-ray diffraction analysis was used to confirm the three-dimensional conformation of **4** and showed that the molecules form pairs of dimers in an inversion related alternate head-to-tail molecular formation in the bc plane. Hirshfeld surface analysis revealed the important contacts within the crystal structure that led to the molecular packing and observed conformation of **4**. Using DFT calculations, the optimised molecular structure (**4a**) free from the influence of the crystal field, FMO and electronic properties were deduced. Results indicated that the conformation of the crystal structure was significantly influenced by the crystal packing forces and confirmed that **4a** is the optimal conformer for docking analysis. Docking experiments showed that **4a** forms important binding interactions with amino acids within the active site of the 11β-HSD1 enzyme and presented with a binding affinity similar to a known potent inhibitor. These results have therefore provided valuable structural information of compound **4** that may be used in the further development of this compound or derivatives thereof as potential therapeutic agents.

## Materials and Methods

### Synthesis of 5-(adamantan-1-yl)-3-[(4-chlorobenzyl)sulfanyl]-4-methyl-4*H*-1,2,4-triazole (4)

The synthesis method followed is similar to previously described by our group^28^ with some minor optimization steps. 4-Chlorobenzyl chloride (322 mg, 2 mmol) and anhydrous potassium carbonate (276 mg, 2 mmol) were added to a solution of 5-(adamantan-1-yl)-4-methyl-4*H*-1,2,4-triazole-3-thiol **3** (499 mg, 2 mmol) in DMF (8 mL), and the mixture was stirred at room temperature for 6 hours. Water (20 mL) was then gradually added to the reaction mixture with stirring for 30 minutes. The precipitated crude product was filtered, washed with water (20 mL), dried and crystallized from aqueous ethanol to yield 688 mg (92%) compound **4** (C_20_H_24_ClN_3_S) as colorless prism crystals. M.p.: 175–177 °C. Single crystals suitable for X-ray analysis were obtained by slow evaporation of an ethanolic solution at room temperature. ^1^H NMR (DMSO-d_6_, 700.17 MHz): δ 1.72–1.77 (m, 6 H, adamantane-H), 2.0 (s, 6 H, adamantane-H), 2.04 (s, 3 H, adamantane-H), 3.47 (s, 3 H, CH_3_), 4.26 (s, 2 H, benzylic CH_2_), 7.26 (d, 2 H, Ar-H, *J* = 8.3 Hz), 7.35 (d, 2 H, Ar-H, *J* = 8.3 Hz). ^13^C NMR (DMSO-d_6_, 176.08 MHz): δ 32.67 (CH_3_), 27.99, 34.94, 37.11, 40.49 (adamantane-C), 39.35 (benzylic-CH_2_), 128.84, 131.21, 132.50, 137.03 (Ar-C), 150.34, 131.26 (Triazole-C).

### X-ray crystallography

Single-crystal X-ray diffraction data was collected at 160(1) K on a Rigaku OD XtaLAB Synergy, Dualflex, Pilatus 200 K diffractometer using a single wavelength X-ray source (Cu Kα radiation: λ = 1.54184 Å)^43^ from a micro-focus sealed X-ray tube and an Oxford liquid-nitrogen Cryostream cooler. The selected suitable single crystal was mounted using polybutene oil on a flexible loop fixed on a goniometer head and immediately transferred to the diffractometer. Pre-experiment, data collection, data reduction and analytical absorption correction^44,45^ were performed with the program suite *CrysAlisPro*^44^ using *Olex2*^46^. The structure was solved with the SHELXT^47^ small molecule structure solution program and refined with the SHELXL 2018/3 program package^48^ by full-matrix least-squares minimization on F2. PLATON^49^ was used to check the result of the X-ray analysis. The crystal data and refinement parameters are shown in Table 4.

**Table 4:** Single crystal X-ray crystallographic data of **4**.

Data	Compound 4
Formula	C_20_H_24_ClN_3_S
Formula weight	373.93
Temperature (K)	160
Crystal system, Space group	Monoclinic, *P*2_1_/n
*a*, *b*, *c* (Å)	6.61380(10), 13.3456(2), 20.9061(4)
ß (°)	96.361(1)
V (Å^3^)	1833.92(5)
*Z*	4
Radiation type	CuKα (λ = 1.54184)
*μ* (mm^−1^)	2.954
No. of reflections	13268
No. of unique reflections/obs. reflections	3708/3107
No. of parameters	227
No. of restraints	0
Δρ_max_, Δρ_min_ (e Å^−3^)	+0.39, −0.39
*T*_min_, *T*_max_	0.706, 0.875
*R*_int_	0.0353
Crystal size (mm)	0.18 × 0.08 × 0.05
R[*F*^2^ > 2σ(*F*^2^)], wR(*F*^2^), S	0.0379, 0.0865, 1.020
CCDC number	1918378

A CIF file containing complete information of the studied structure was deposited with CCDC, deposition number 1918378 and is freely available upon request from the Director, CCDC, 12 Union Road, Cambridge CB2 1EZ, UK (Fax: + 44-1223-336033; e-mail: deposit@ccdc.cam.ac.uk) or from the following website: www.ccdc.cam.ac.uk/data_request/cif.

### Hirshfeld surface analysis

The Hirshfeld surfaces and 2D fingerprint plots were generated using Crystal Explorer 3.1^50^. The X-ray single-crystal crystallographic information file of **4** was used as input file. The TONTO application within Crystal Explorer was used to calculate the electrostatic potential surface map using B3LYP/6-311G(d,p) ± 0.03 a.u.

#### Quantum chemical calculations

A crystal unit within the crystal structure of the **4** was selected as the starting structure for the DFT calculations. Structural optimization was performed using the Gaussian09 program package^31^, in the same manner as previously reported by our group^26^. Energy minima calculations were carried out using the Becke’s three parameters Lee-Yang-Parr exchange correlation functional (B3LYP). This calculation combines Becke’s hybrid exchange functional^32^ with Lee, Yang and Parr’s gradient-correlation functional using the base set of 6-311 G++(d,p)^33^. Previous studies have shown a good correlation between gas phase calculations and crystal structures, therefore no solvent corrections were made^34^. The geometry was optimized and the DFT optimized molecule (**4a**) was generated. Vibration analysis showed no negative Eigen values indicating that the optimized structure (**4a**) represents a minimum on the potential energy surface. A complete list of coordinates, bond lengths and bond angles for both the experimental crystal structure and **4a** are given in the Supplementary materials (Tables S1–S3 and Fig. S1). For the optimized structure, the HOMO, LUMO and atomic net charges were drawn using Avogadro 1.2.0^51,52^.

#### Molecular docking studies

The molecular docking studies were performed using the human 11β-HSD1 structure (Protein Data Bank I.D.: 4C7J). The docking method employed is similar to that reported by our group for studies on protease- and neuronal nitric oxide synthase enzymes^26,53^. The Molecular Operating Environment (MOE) 2018 software suite^41^ was used for docking studies with the following protocol. (1) The enzyme protein structure was checked for missing atoms, bonds and contacts. (2) Removal of water molecules, 3D protonation and energy minimization was carried out with parameters, force field: MMFF94X + solvation, gradient: 0.05, chiral constraint and current geometry. This minimized structure was used as enzyme for docking analysis. (3) The DFT B3LYP/6-311++G (d,p) optimized structure 4**a** was saved as a pdb file and imported into the MOE database. (4) Conformer **4a** was subsequently docked within the human 11β-HSD1 active site using the MOE Dock application. The active site was selected based on the proximity of the co-crystallized ligand, 4YQ, with the help of the MOE Site Finder tool. The docking algorithm, which was chosen for these experiments, was based on induced fit docking to allow for flexible interactions of the test ligand with the protein. (5) The best binding pose of the title compound was visually inspected and the interactions with the binding pocket residues were analyzed. The selected parameters that were used to calculate the score and interaction of the ligand molecule with the 11β-HSD1 enzyme were; Rescoring function: London dG, Placement: Triangle matcher, Retain: 30, Refinement: Force field, Rescoring 2: London dG. The build-in scoring function of MOE, S-score, was used to predict the binding affinity (kcal/mol) of **4a** with the enzyme protein active site after docking.

## Supplementary information

Supplementary information 
