# Supplementary material for: Crystal structure, Hirshfeld surface analysis and DFT studies of 5-(adamantan-1-yl)-3-[(4-chlorobenzyl)sulfanyl]-4-methyl-4H-1,2,4-triazole, a potential 11β-HSD1 inhibitor
Source: Sci Rep. 2019 Dec 24;9:19745. doi: 10.1038/s41598-019-56331-z (PMC6930263; doi:10.1038/s41598-019-56331-z)
Supplement: Supplementary file 1 — Supplementary information [file 41598_2019_56331_MOESM1_ESM.pdf]

Supplementary Materials: Crystal structure, Hirshfeld surface analysis and DFT studies of 5-(adamantan-1-yl)-3-[(4-chlorobenzyl)sulfanyl]-4-methyl-4*H*-1,2,4-triazole, a potential 11β-HSD1 inhibitor

Lamya H. Al-Wahaibi, Jacques Joubert, Olivier Blacque, Nora H. Al-Shaalan, and Ali A. El-Emam

Table S1: The calculated bond distances (Å) of **4a** compared to the experimental data of the title compound.

		Bond Length	
Atom1	Atom2	Experimental	Theoretical
C1	C2	1.376	1.3900
C1	C6	1.3747	1.3914
C1	Cl	1.7451	1.7605
C2	C3	1.3862	1.3922
C3	C4	1.3842	1.3993
C4	C5	1.3900	1.398
C4	C7	1.5055	1.5051
C5	C6	1.3876	1.3917
C7	S1	1.8321	1.8422
C8	N1	1.3698	1.3661
C8	N2	1.3094	1.3031
C8	S1	1.7519	1.7761
C9	C11	1.5047	1.5157
C9	N1	1.3740	1.4044
C9	N3	1.3157	1.3031
C10	N1	1.4639	1.4587
C11	C12	1.5467	1.5563
C11	C16	1.5403	1.5512
C11	C19	1.5461	1.5518
C12	C13	1.5331	1.5452
C13	C14	1.5312	1.5414
C13	C17	1.5335	1.5376
C14	C15	1.5307	1.5402
C15	C16	1.5338	1.5410
C15	C20	1.5338	1.5362
C17	C18	1.5303	1.5390
C18	C19	1.5313	1.5397
C18	C20	1.5280	1.5405
N2	N3	1.3800	1.3879

Table S2: The calculated bond angles (Å) **4a** compared to the experimental data of the title compound.

			Bond angle	
Atom1	Atom2	Atom3	Experimental	Theoretical
C2	C1	C6	121.53	121.15
C2	C1	Cl1	119.39	119.47
C6	C1	Cl1	119.08	119.38
C1	C2	C3	118.87	118.94
C2	C3	C4	120.93	121.25
C3	C4	C5	119.05	118.45
C3	C4	C7	121.42	120.54
C5	C4	C7	119.51	121.02
C4	C5	C6	120.41	121.08
C1	C6	C5	119.22	119.13
C4	C7	S1	107.11	108.71
N1	C8	N2	110.83	111.83
N1	C8	S1	125.05	121.43
N2	C8	S1	124.05	126.58
C11	C9	N1	127.84	127.63
C11	C9	N3	122.84	123.40
N1	C9	N3	109.32	108.94
C9	C11	C12	111.98	112.88
C9	C11	C16	111.34	111.04
C9	C11	C19	108.14	108.30
C12	C11	C16	109.75	108.82
C12	C11	C19	107.89	107.09
C16	C11	C19	107.56	108.30
C11	C12	C13	109.96	110.62
C12	C13	C14	109.84	109.47
C12	C13	C17	109.60	109.76
C14	C13	C17	109.33	109.29
C13	C14	C15	109.58	109.55
C14	C15	C16	109.46	109.47
C14	C15	C20	109.76	109.59
C16	C15	C20	109.42	109.51
C13	C17	C18	109.24	109.09
C17	C18	C19	109.69	109.62
C17	C18	C20	109.56	109.36
C19	C18	C20	109.67	109.99
C11	C19	C18	110.64	110.86
C15	C20	C18	109.00	109.18
C8	N1	C9	104.71	103.81
C8	N1	C10	124.56	124.96

C9	N1	C10	130.71	131.18
C8	N2	N3	106.74	106.41
C9	N3	N2	108.40	109.01
C7	S1	C8	102.14	98.08

Theoretical Calculations of 4a

According to the crystal structure, a crystal unit was selected as the initial structure, while DFT-B3LYP/6-311G++(d,p) methods in Gaussian09 was used to optimize the structure of the title compound (**4a**, Figure S1).[1] No solvent corrections were made with these calculations. Vibration analysis showed that the optimized structure indeed represents a minimum on the potential energy surface (no negative eigenvalues). This conformer showed an energy value of (27.973 kcal/mol).

Table S3: DFT Optimised Cartesian Coordinates (Å) for compound **4a**.

opt B3LYP/6-311++G(d,p)

0 1

C	-6.779	-0.37	-0.219
C	-5.832	-1.138	-0.886
H	-6.117	-2.076	-1.349
C	-4.519	-0.68	-0.953
H	-3.774	-1.278	-1.47
C	-4.142	0.532	-0.364
C	-5.119	1.285	0.294
H	-4.853	2.234	0.749
C	-6.436	0.842	0.372
H	-7.192	1.429	0.882
C	-2.717	1.01	-0.443
H	-2.633	2.094	-0.367
H	-2.217	0.69	-1.354
C	-0.143	0.752	0.438
C	1.997	0.609	0.045
C	0.921	-1.219	1.561
H	0.687	-2.127	1
H	0.149	-1.088	2.321
H	1.873	-1.335	2.074
C	3.446	0.171	-0.031
C	4.278	0.615	1.207
H	4.216	1.705	1.304
H	3.867	0.192	2.131
C	5.751	0.172	1.06

H	6.303	0.488	1.954
C	5.815	-1.362	0.924
H	6.86	-1.688	0.839
H	5.41	-1.845	1.823
C	5.027	-1.802	-0.324
H	5.066	-2.893	-0.419
C	3.555	-1.369	-0.181
H	3.124	-1.872	0.69
H	2.98	-1.695	-1.057
C	6.366	0.819	-0.192
H	6.36	1.912	-0.096
H	7.417	0.518	-0.293
C	5.562	0.384	-1.43
H	5.979	0.859	-2.327
C	4.096	0.829	-1.277
H	3.521	0.57	-2.172
H	4.033	1.915	-1.184
C	5.635	-1.148	-1.574
H	6.676	-1.47	-1.699
H	5.096	-1.473	-2.473
Cl	-8.443	-0.937	-0.124
N	0.941	-0.033	0.712
N	0.174	1.773	-0.307
N	1.534	1.665	-0.562
S	-1.775	0.296	0.97

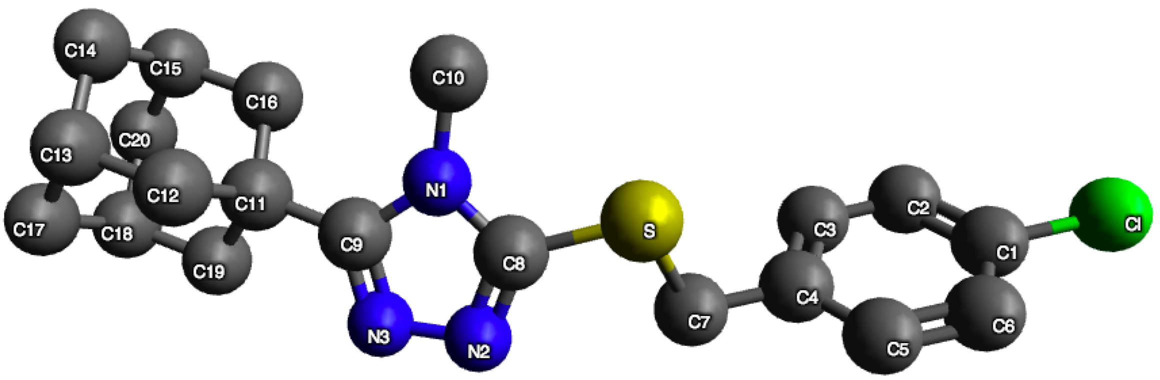

Figure S1: DFT Optimized geometry of compound **4a** (B3LYP/6-311++G(d,p)).

Theoretical Calculations of 4b

A crystal unit was selected from the crystal structure and imported into Molecular Operating Environment 2018.01 (MOE) software.[2] MOE was used to fix the C8-S1 bond orthogonally. Where after the structure was optimized (**4b**) with DFT-B3LYP/6-311G++(d,p) methods using the Gaussian09 plug-in within MOE as described for **4a**.[1] By fixing the C8-S1, a conformation very similar to the single crystal structure was generated (**4b**) as indicated in Figure S3 (RMSD = 0.221 Å). This conformer showed an energy value of (47.495 kcal/mol).

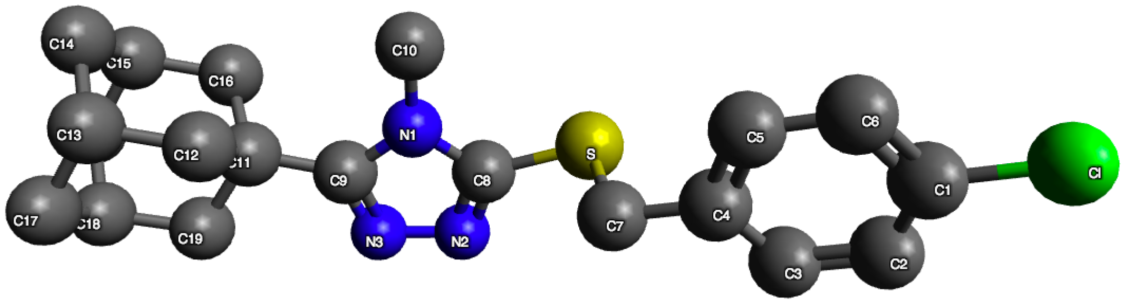

Figure S2: DFT Optimized geometry of compound **4b** (B3LYP/6-311++G(d,p)).

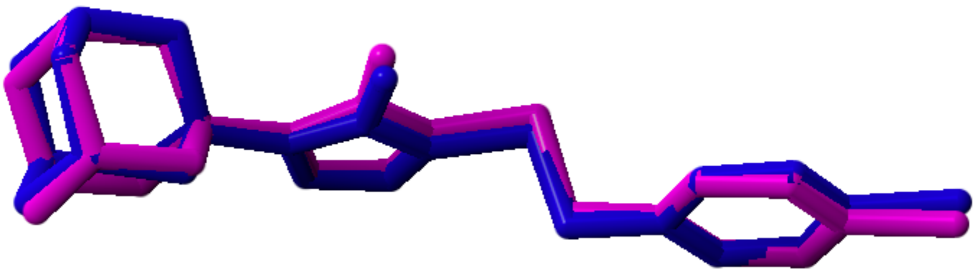

Figure S3: Atom-by-atom superimposition of the DFT optimized compound (**4b**, magenta) on the X-ray structure (blue) of the title compound (RMSD = 0.221 Å, performed using YASARA version 18.11.21 (YASARA Biosciences GmbH)).

References

1. Frisch, M. J., Trucks, G. W., Schlegel, H. B., Scuseria, G. E., Robb, M. A., Cheeseman, J. R., Scalmani, G., Barone, V., Mennucci, B., Petersson, G. A. *et al.* Gaussian 09, Gaussian, Inc: Wallingford, CT, USA (2009).
2. Molecular Operating Environment (MOE), Version 2018.01. http://www.chemcomp.com.

checkCIF/PLATON report

Structure factors have been supplied for datablock(s) 4

THIS REPORT IS FOR GUIDANCE ONLY. IF USED AS PART OF A REVIEW PROCEDURE FOR PUBLICATION, IT SHOULD NOT REPLACE THE EXPERTISE OF AN EXPERIENCED CRYSTALLOGRAPHIC REFEREE.

No syntax errors found. CIF dictionary Interpreting this report

Datablock: 4

```
Bond precision:  C-C = 0.0027 A          Wavelength=1.54184

Cell:            a=6.6138(1)     b=13.3456(2)       c=20.9061(4)
                 alpha=90        beta=96.361(1)     gamma=90
Temperature:     160 K

                Calculated                 Reported
Volume          1833.92(5)                 1833.92(5)
Space group     P 21/n                     P 1 21/n 1
Hall group      -P 2yn                     -P 2yn
Moiety formula  C20 H24 Cl N3 S            C20 H24 Cl N3 S
Sum formula     C20 H24 Cl N3 S            C20 H24 Cl N3 S
Mr              373.93                     373.93
Dx,g cm-3       1.354                      1.354
Z               4                          4
Mu (mm-1)       2.954                      2.954
F000            792.0                      792.0
F000'           796.50
h,k,lmax        8,16,26                    8,16,26
Nref            3753                       3708
Tmin,Tmax       0.753,0.863                0.706,0.875
Tmin'           0.588

Correction method= # Reported T Limits: Tmin=0.706 Tmax=0.875
AbsCorr = ANALYTICAL

Data completeness= 0.988          Theta(max)= 74.471

R(reflections)= 0.0379( 3107)     wR2(reflections)= 0.0865( 3708)

S = 1.020                  Npar= 227
```

The following ALERTS were generated. Each ALERT has the format
test-name_ALERT_alert-type_alert-level.
Click on the hyperlinks for more details of the test.

Alert level C

```
PLAT906_ALERT_3_C Large K Value in the Analysis of Variance ......      2.621 Check
PLAT911_ALERT_3_C Missing FCF Refl Between Thmin & STh/L=    0.600         20 Report
```

Alert level G

```
PLAT912_ALERT_4_G Missing # of FCF Reflections Above STh/L=  0.600         26 Note
PLAT978_ALERT_2_G Number C-C Bonds with Positive Residual Density.         12 Info
```

```
   0 ALERT level A = Most likely a serious problem - resolve or explain
   0 ALERT level B = A potentially serious problem, consider carefully
   2 ALERT level C = Check. Ensure it is not caused by an omission or oversight
   2 ALERT level G = General information/check it is not something unexpected

   0 ALERT type 1 CIF construction/syntax error, inconsistent or missing data
   1 ALERT type 2 Indicator that the structure model may be wrong or deficient
   2 ALERT type 3 Indicator that the structure quality may be low
   1 ALERT type 4 Improvement, methodology, query or suggestion
   0 ALERT type 5 Informative message, check
```

It is advisable to attempt to resolve as many as possible of the alerts in all categories. Often the minor alerts point to easily fixed oversights, errors and omissions in your CIF or refinement strategy, so attention to these fine details can be worthwhile. In order to resolve some of the more serious problems it may be necessary to carry out additional measurements or structure refinements. However, the purpose of your study may justify the reported deviations and the more serious of these should normally be commented upon in the discussion or experimental section of a paper or in the "special_details" fields of the CIF. checkCIF was carefully designed to identify outliers and unusual parameters, but every test has its limitations and alerts that are not important in a particular case may appear. Conversely, the absence of alerts does not guarantee there are no aspects of the results needing attention. It is up to the individual to critically assess their own results and, if necessary, seek expert advice.

Publication of your CIF in IUCr journals

A basic structural check has been run on your CIF. These basic checks will be run on all CIFs submitted for publication in IUCr journals (*Acta Crystallographica*, *Journal of Applied Crystallography*, *Journal of Synchrotron Radiation*); however, if you intend to submit to *Acta Crystallographica Section C* or *E* or *IUCrData*, you should make sure that full publication checks are run on the final version of your CIF prior to submission.

Publication of your CIF in other journals

Please refer to the *Notes for Authors* of the relevant journal for any special instructions relating to CIF submission.

PLATON version of 03/05/2019; check.def file version of 29/04/2019

Datablock 4 - ellipsoid plot

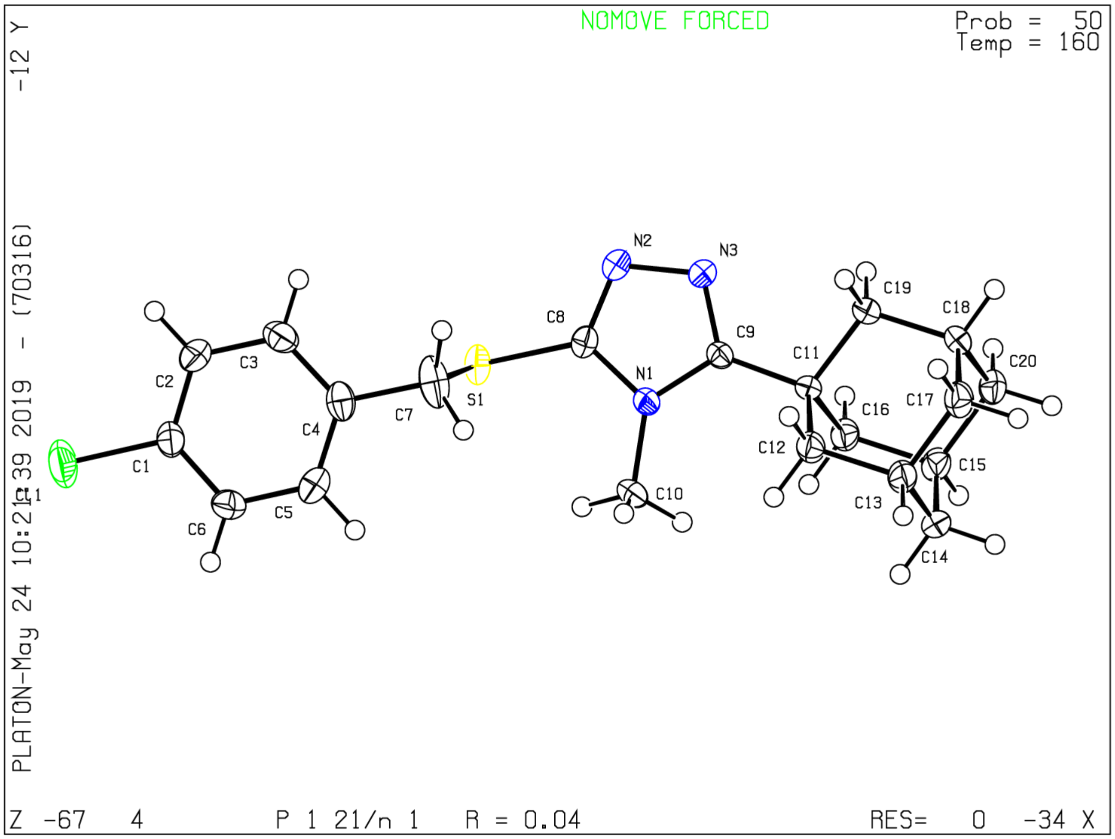

H-NMR

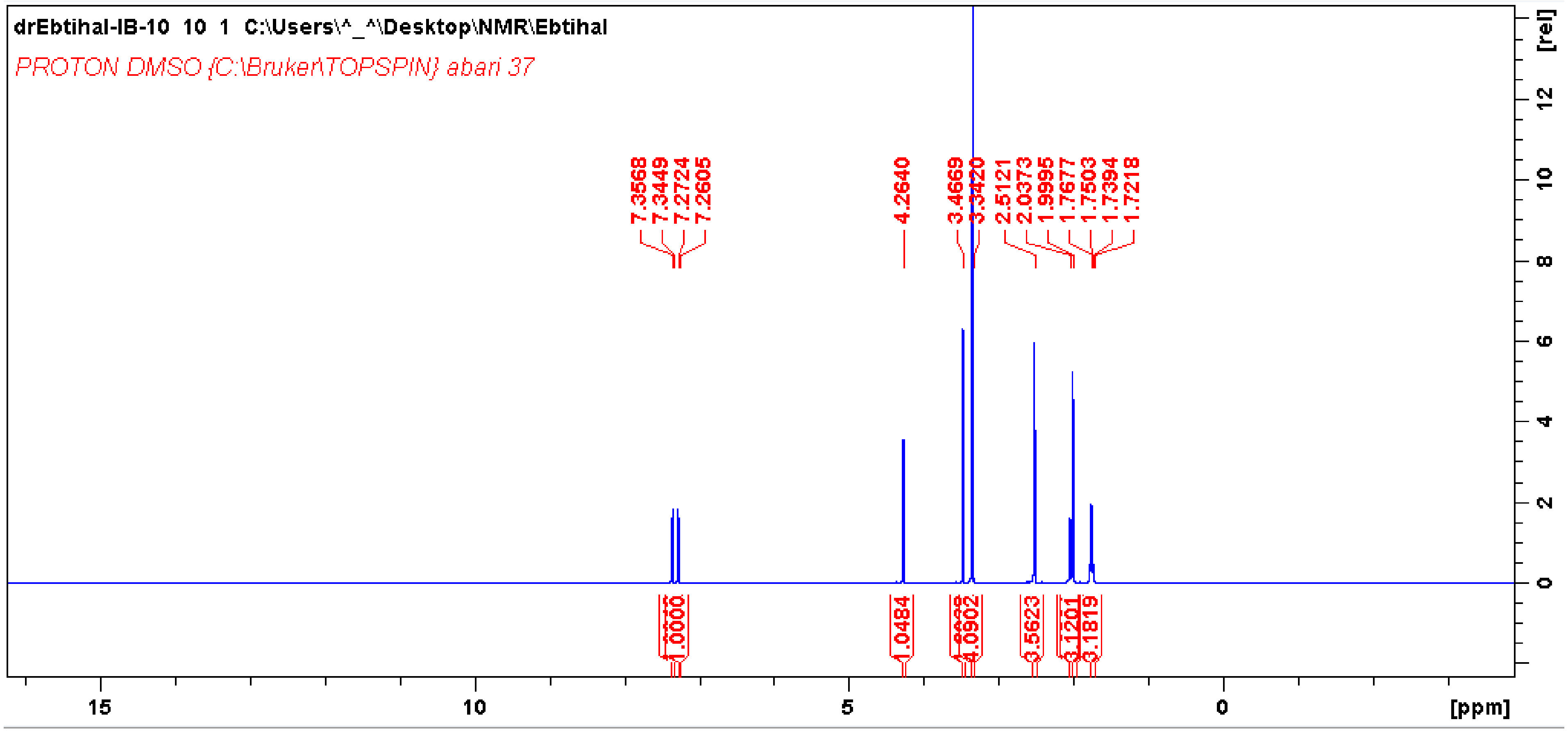

drEbtihal-IB-10 10 1 C:\Users\^_^\Desktop\NMR\Ebtihal
PROTON DMSO {C:\Bruker\TOPSPIN} abari 37
7.3568
7.3449
7.2724
7.2605
4.2640
3.4669
3.3420
2.5121
2.0373
1.9995
1.7677
1.7503
1.7394
1.7218
1.0000
1.0484
4.0902
3.5623
3.1201
3.1819
15
10
5
0
[ppm]
0
2
4
6
8
10
12
[rel]

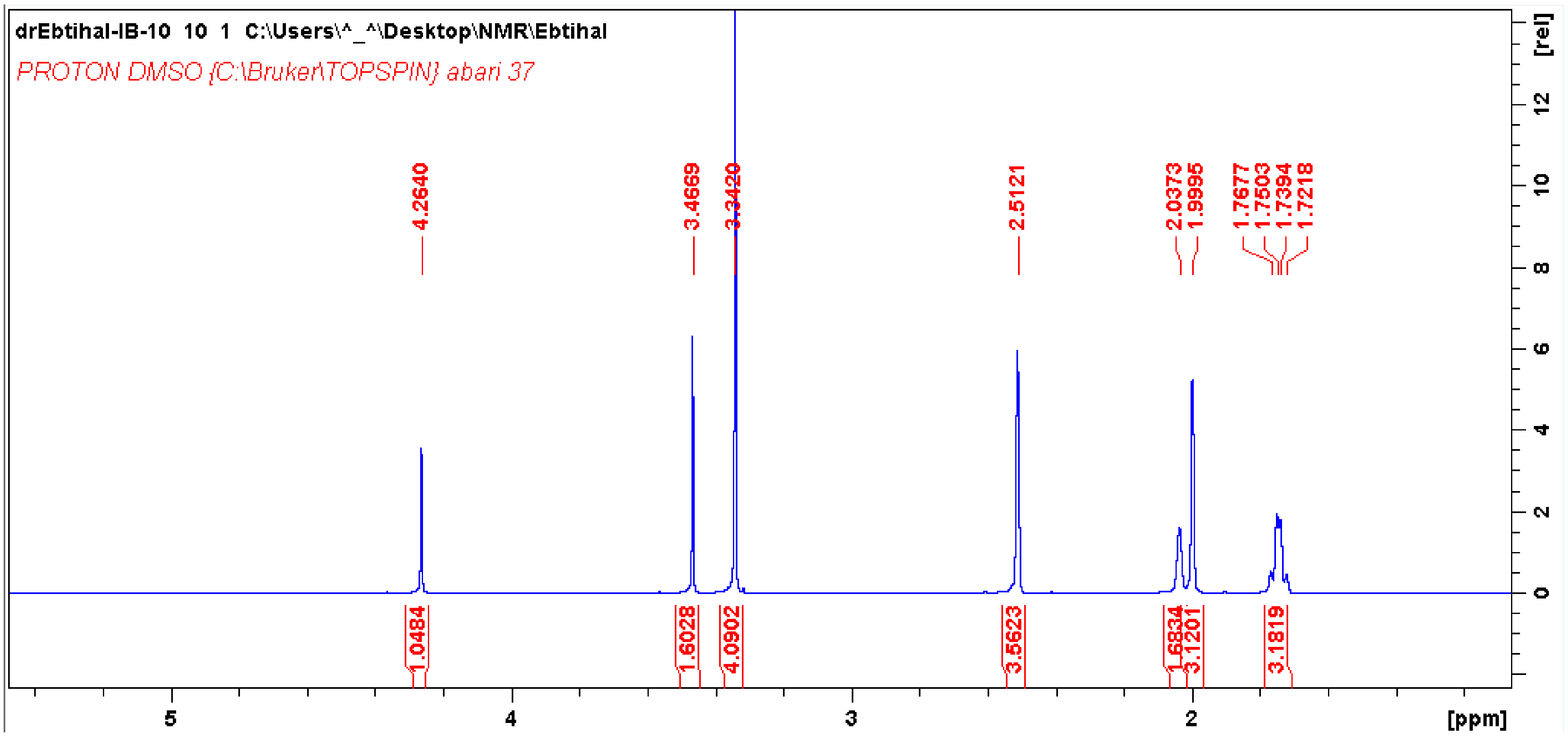
drEbtihal-IB-10 10 1 C:\Users\^_^\Desktop\NMR\Ebtihal
PROTON DMSO {C:\Bruker\TOPSPIN} abari 37
4.2640
3.4669
3.3420
2.5121
2.0373
1.9995
1.7677
1.7503
1.7394
1.7218
1.0484
1.6028
4.0902
3.5623
1.6834
3.1201
3.1819
[rel]
[ppm]

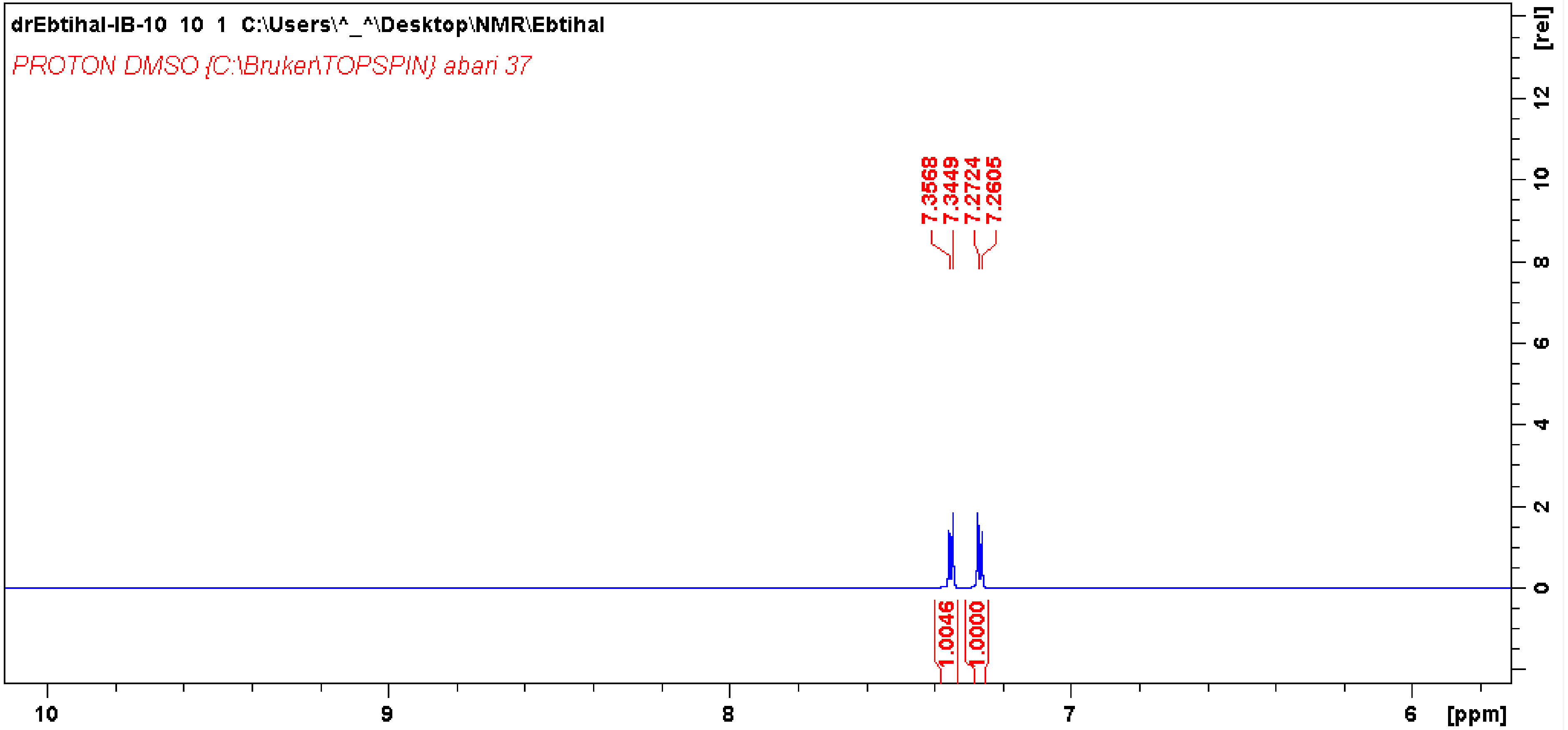
drEbtihal-IB-10 10 1 C:\Users\^_^\Desktop\NMR\Ebtihal
PROTON DMSO {C:\Bruker\TOPSPIN} abari 37
7.3568
7.3449
7.2724
7.2605
1.0046
1.0000
10
9
8
7
6
[ppm]
[rel]
12
10
8
6
4
2
0

700 MHz

Solvent: DMSO

Hanan Alshibl, TopSpin [Academic license]

C-NMR

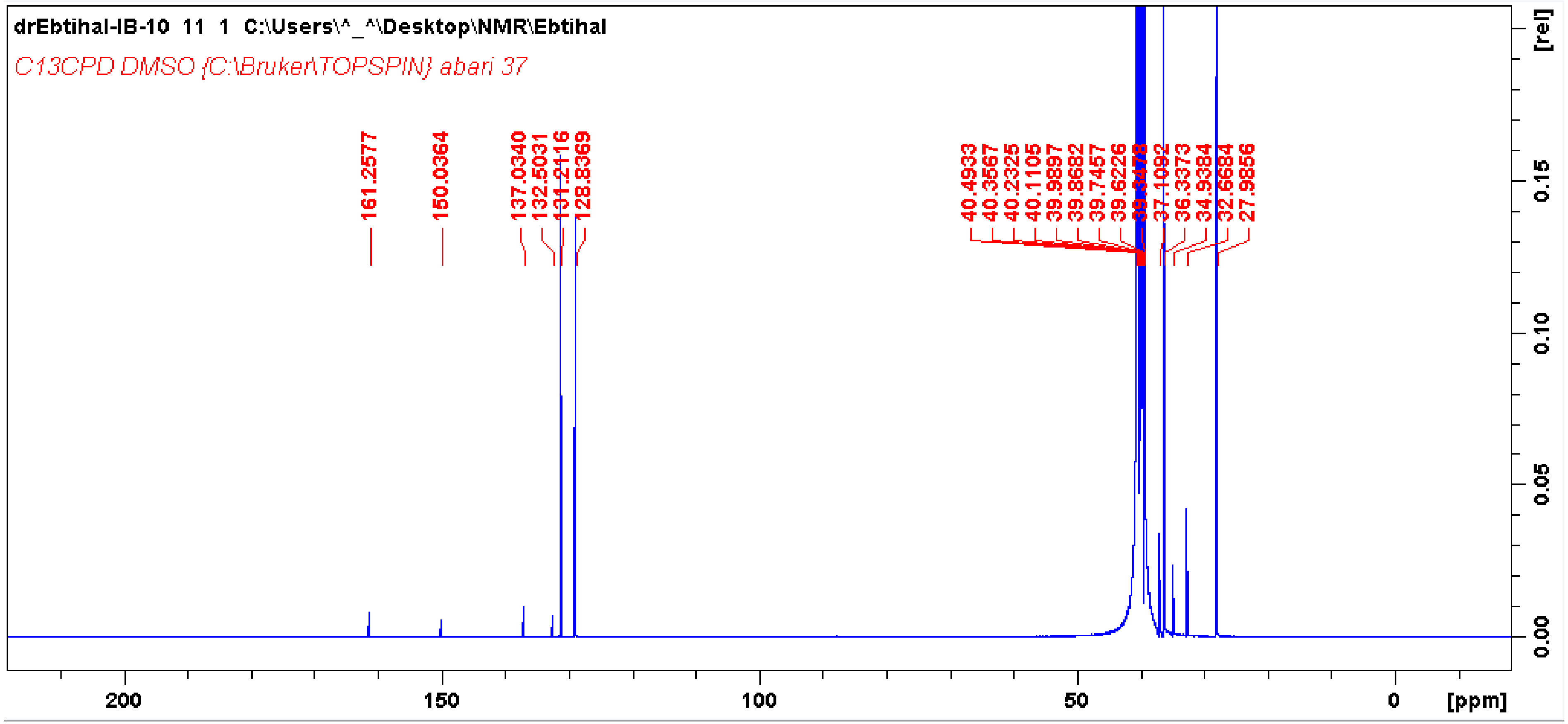
drEbtihal-IB-10 11 1 C:\Users\^_^\Desktop\NMR\Ebtihal
C13CPD DMSO {C:\Bruker\TOPSPIN} abari 37
161.2577
150.0364
137.0340
132.5031
131.2116
128.8369
40.4933
40.3567
40.2325
40.1105
39.9897
39.8682
39.7457
39.6226
37.1092
36.3373
34.9384
32.6684
27.9856
[rel]
0.15
0.10
0.05
0.00
200
150
100
50
0
[ppm]

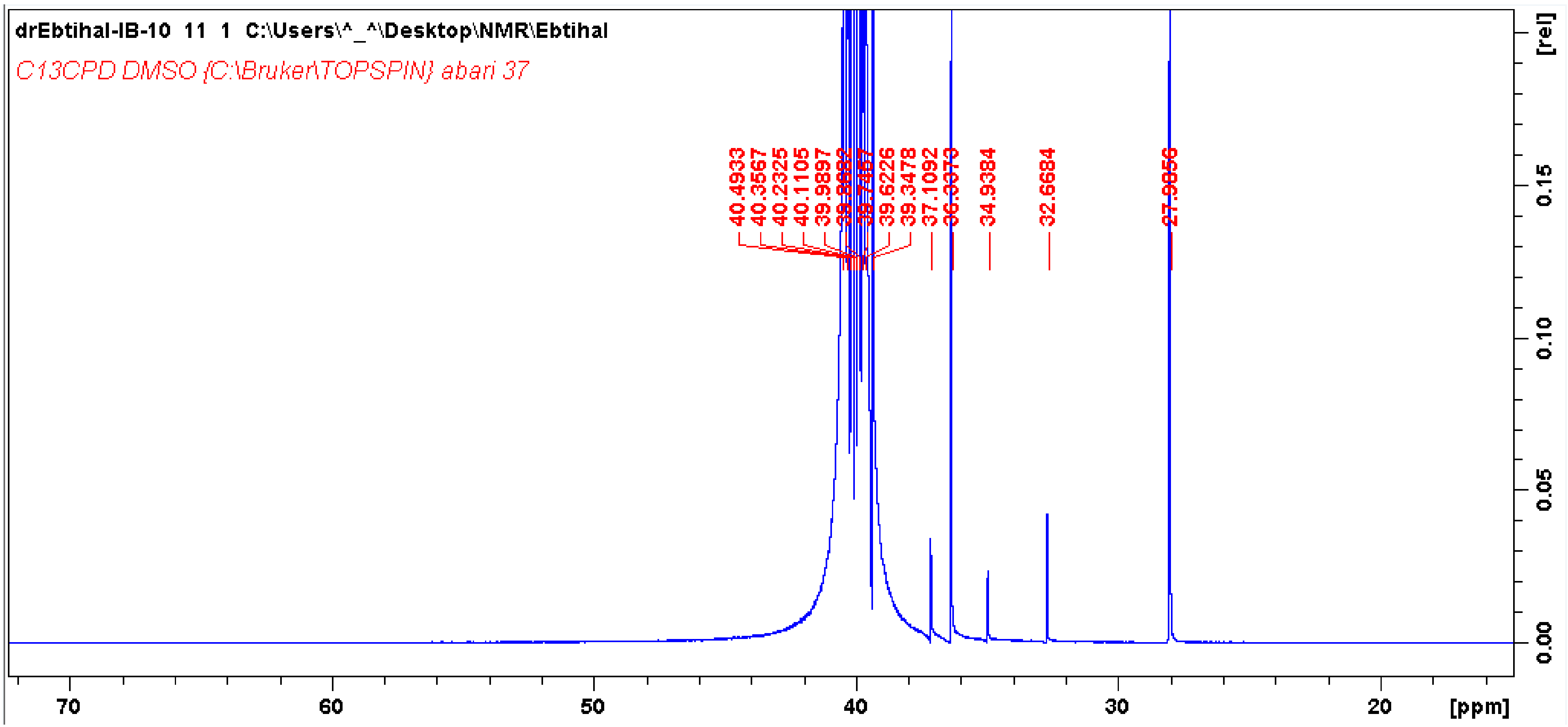
drEbtihal-IB-10 11 1 C:\Users\^_^\Desktop\NMR\Ebtihal
C13CPD DMSO {C:\Bruker\TOPSPIN} abari 37
40.4933
40.3567
40.2325
40.1105
39.9897
39.8682
39.7457
39.6226
39.3478
37.1092
36.3373
34.9384
32.6684
27.9856
[rel]
0.15
0.10
0.05
0.00
70
60
50
40
30
20
[ppm]

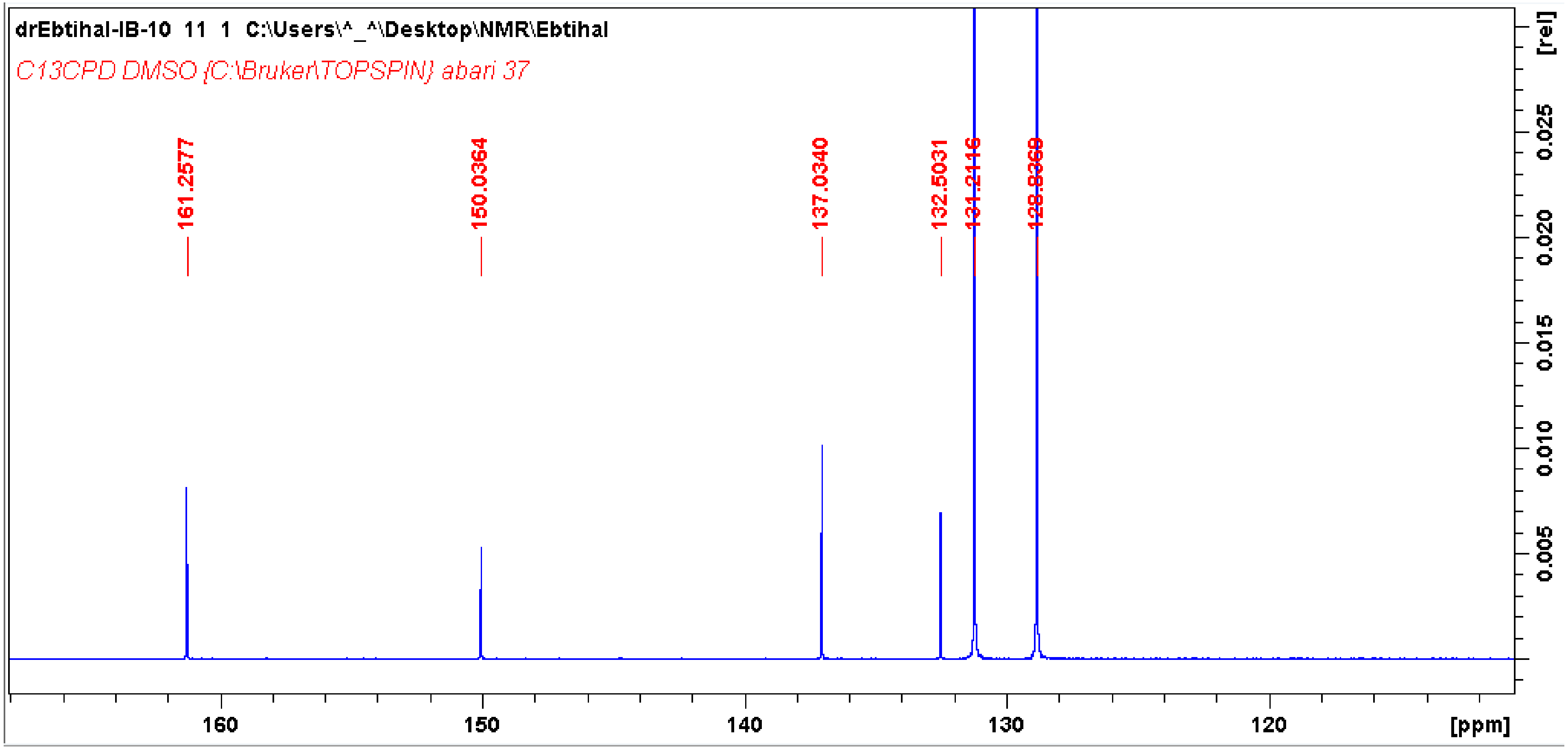

drEbtihal-IB-10 11 1 C:\Users\^_^\Desktop\NMR\Ebtihal
C13CPD DMSO {C:\Bruker\TOPSPIN} abari 37
161.2577
150.0364
137.0340
132.5031
131.2116
128.8369
[rel]
0.025
0.020
0.015
0.010
0.005
160
150
140
130
120
[ppm]
